# *In situ* Generated Ru(0)-HRO@Na-β From Hydrous Ruthenium Oxide (HRO)/Na-β: An Energy-Efficient Catalyst for Selective Hydrogenation of Sugars

**DOI:** 10.3389/fchem.2020.525277

**Published:** 2020-11-25

**Authors:** Sreedhar Gundekari, Heena Desai, Krishnan Ravi, Joyee Mitra, Kannan Srinivasan

**Affiliations:** ^1^Inorganic Materials and Catalysis Division, CSIR-Central Salt and Marine Chemicals Research Institute, Council of Scientific and Industrial Research (CSIR), Bhavnagar, India; ^2^Academy of Scientific and Innovative Research, Council of Scientific and Industrial Research (CSIR)-Central Salt and Marine Chemicals Research Institute, Bhavnagar, India; ^3^Thermo-Chemical Conversion Division, Sardar Patel Renewable Energy Research Institute (SPRERI), Anand, India

**Keywords:** sugars, sugar alcohol, hydrogenation, hydrous ruthenium oxide, *in situ* reduction, recyclable catalyst

## Abstract

A green process for the hydrogenation of sugars to sugar alcohols was designed in aqueous medium using hydrous ruthenium oxide (HRO) as a pre-catalyst supported on Na-β zeolite. Under optimized reaction conditions, sugars such as xylose, glucose, and mannose converted completely to the corresponding sugar alcohols xylitol, sorbitol, and mannitol with 100% selectivity. The pre-catalyst (HRO) is converted *in situ* to active Ru(0) species during the reaction under H_2_, which is responsible for the hydrogenation. The catalyst was recyclable up to five cycles with no loss in activity. The reduction of HRO to the active Ru(0) species is dependent on the reaction temperature and H_2_ pressure. Ru(0) formation increased and consequently an increased hydrogenation of sugars was observed with an increase in reaction temperature and hydrogen pressure. Further, *in situ* generation of Ru(0) from HRO was assessed in different solvents such as water, methanol, and tetrahydrofuran; aqueous medium was found to be the most efficient in reducing HRO. This work further demonstrates the use of supported HRO as an efficient pre-catalyst for biomass-based hydrogenation reactions.

## Introduction

Lignocellulosic biomass is an important raw material for the production of fuels, polymer, and chemical intermediates. Biomass is renewable, unlike fossil resources, and its conversion maintains the CO_2_ level in the atmosphere. Their usage is also beneficial to the rural economy (Werpy et al., 2004; Climent et al., 2011b; Serrano-Ruiz et al., 2011; Vennestrøm et al., 2011; Melero et al., 2012). The Department of Energy (DOE) and universities of Europe listed important building blocks from such lignocellulosic biomass that includes polyols in addition to carboxylic acids, phenolic, and furan compounds (Lange et al., 2012; Kelkar et al., 2014; Sheldon, 2014; Teong et al., 2014). Sugars derived from cellulose and hemicellulose of lignocellulosic biomass are used for the preparation of sugar alcohols by selective hydrogenation, and several industries worldwide are interested in this conversion. The global consumption of sugar alcohols is estimated to be 1.6 million metric tons in 2017 and is projected to reach 1.9 million metric tons by 2022 at a CAGR of 3.4% (Climent et al., 2011a; Luterbacher et al., 2014).

Sugar alcohols such as sorbitol, xylitol, and mannitol are commonly available low-calorie sweeteners and are used in various industries like food, cosmetics, and pharmaceuticals. The sugar alcohols are non-toxic, non-carcinogenic, and non-hygroscopic. Thus, these could be safely consumed by diabetic patients (Grembecka, 2015). Moreover, sorbitol is the starting material for the production of ascorbic acid (vitamin C) and also is a precursor for hexane fuel (Corma et al., 2007; Alonso et al., 2012). Aside from their widespread use as sweeteners, the sorbitol-derived isosorbide and anhydro-sugars are industrially relevant as precursors for the preparation of PET like polymers such as polyethylene isosorbide terephthalates. The xylitol-derived xylaric, xylonic acid, and the mixture of hydroxyl furans can open up new opportunities in polymer preparation. The specific hydrogenolysis (C-C and C-O) of sorbitol and xylitol results in polyols like propylene glycol, ethylene glycol, and glycerol (Supplementary Figure 1). Controlled hydrogenolysis of sugar alcohols results in lactic acid, which is largely used in polylactate production (Bozell and Petersen, 2010; Gallezot, 2012; Kobayashi and Fukuoka, 2013; Isikgor and Becer, 2015; Zada et al., 2017).

The hydrogenation of sugars to sugar alcohols has been extensively studied with homogeneous and heterogeneous catalysts, among which heterogeneous Ni- and Ru-based catalysts are found to be more effective (Corma et al., 2007; Alonso et al., 2012; Chatterjee et al., 2015; Zhang et al., 2016; Zada et al., 2017). This hydrogenation is industrially practiced mainly with a Raney® Ni catalyst under aqueous basic medium and encounters a problem of Ni leaching. Many elements have been incorporated in Raney® Ni to improve the stability and enhance the catalytic activity (Wisniak et al., 1974; Chao and Huibers, 1982). Mo-, P-, Cr-, and Fe-promoted Raney® Ni catalyst showed lesser deactivation for the conversion of glucose to sorbitol and xylose to xylitol (Li et al., 2000; Mikkola et al., 2000; Kusserow et al., 2003). Ni–B/SiO_2_ amorphous catalyst (prepared by chemical reduction with KBH_4_) rendered a good conversion of glucose as compared to conventional Raney® Ni (Li et al., 2002). Morales et al. discussed a mixed metal oxide catalyst La_1−x_Ce_*x*_Al_0.18_Ni_0.82_O_3_ (*x* = 0.0, 0.1, 0.5, 0.7) for xylose-to-xylitol conversion at 100°C, 25 bar H_2_ for 5 h and achieved 100% conversion with moderate selectivity (Morales et al., 2016).

To counter the leaching issues with Ni catalysts, Ru-based catalysts have also been employed for this hydrogenation. The supported Ru catalysts show good catalytic activity, product selectivity, and stability as compared with Ni-based catalysts. Guo et al. reported an ultrafine Ru-B amorphous alloy catalyst for the conversion of glucose to sorbitol. This catalyst was shown to be more active than crystallized Ru-B and Ru powder catalysts (Guo et al., 2003). Ru catalysts employed mainly two supports [i.e., carbon (different forms: activated, foam, and nanotubes) and γ-Al_2_O_3_ under batch and continuous modes at 110–130°C and 20–40 bar H_2_ to yield 95–98% of the desired sugar alcohols (Arena, 1992; Hoffer et al., 2003; Eisenbeis et al., 2009; Sifontes Herrera et al., 2011; Aho et al., 2015; Pham et al., 2016)].

A Ru/NiO-TiO_2_ catalyst reported by Hwang's group resulted in 96% conversion of glucose with 98% selectivity of sorbitol at 120°C, 55 bar H_2_ for 2 h. Complete conversion of mannose with >90% selectivity of mannitol in 4 h and >99% conversion of xylose with >99% selectivity for xylitol in 2 h was also reported (Mishra et al., 2012; Yadav et al., 2012; Mishra and Hwang, 2013). The same research group reported Ru/H-Y zeolite (prepared by NaBH_4_ reduction in ethanol under N_2_) catalyst for sugar hydrogenation showing 98% conversion of xylose and 98% selectivity for xylitol and >98% selectivity of sorbitol with the quantitative conversion of glucose under 55 bar of H_2_ at 120°C for 2–3 h (Mishra et al., 2013, 2014). Ru supported on amine functionalized nanoporous polymer (AFPS) was effective in converting glucose to sorbitol with 98% selectivity at 100°C, 55 bar H_2_ for 2 h (Dabbawala et al., 2016). However, in all these reports, a very high hydrogen pressure was required to enable such high yields.

The Mu group reported Ru/ZSM-5 (obtained from H_2_ reduction) as catalyst for hydrogenation of glucose to sorbitol in 2 h with 99% conversion and selectivity at 120°C, under 40 bar H_2_ (Guo et al., 2014). Zhang et al. screened several catalysts for glucose hydrogenation to sorbitol, including Ru/MCM-41, Pd/C, Ru/C, and Raney® Ni. Among these, the Ru/MCM-41 (obtained from formaldehyde reduction process) catalyst showed highest catalytic activity (complete conversion with >80% selectivity) at 120°C, 30 bar H_2_ for 2 h. However, a decrease in the catalytic activity was observed in subsequent reaction cycles (Zhang et al., 2011). The Shiju group employed Ru on TiO_2_ (calcined at 800–900°C) for the complete conversion of xylose to xylitol with 98% yield at 120°C and 20 bar H_2_ (Hernandez-Mejia et al., 2016).

Generally, the catalysts used for this hydrogenation are reduced metals that require a pre-reduction step before reaction. The step involves additional energy (electricity, H_2_, and manpower) and time, and is often more energy-intensive compared to the catalytic reaction. To minimize the energy requirements for the synthesis of active catalysts used in hydrogenation and hydrodeoxygenation reactions, our group has been working on the *in situ* generated catalysts. Hydrous ruthenium oxide (HRO) is one such efficient pre-catalyst wherein the catalytically active Ru(0) species is generated *in situ* under mild reaction conditions in an aqueous medium that drives the reaction (Gundekari and Srinivasan, 2019). In the present work, HRO is discussed as the pre-catalyst for the hydrogenation of sugar to sugar alcohols. A comparison of the performance of our *in situ* generated catalyst with the reported catalytic systems have been summarized in the Supplementary Table 1.

## Experimental

### Materials and Methods

Sorbitol (≥98%), xylitol (≥99%), mannose (99%), and RuCl_3_.xH_2_O were purchased from Sigma-Aldrich. Xylose (98%), mannitol (99%), and RuO_2_ were procured from Alfa Aesar. The Na-β zeolite was purchased from Zeochem, Switzerland. Glucose, metal salts, and hydrogen (>99.99% purity) were purchased from local vendors in India.

### Catalyst Preparation

#### Hydrous Ruthenium Oxide (HRO)

HRO catalyst was prepared by a simple precipitation method: a solution 0.001 M of RuCl_3_ was added to the appropriate amount of CaCO_3_ aqueous solution and allowed to stand for 1 h without any stirring and heating. pH 7–8 was maintained during the reaction. The obtained precipitate was washed several times with water for the removal of chloride ions (confirmed with AgNO_3_ solution) and dried for 3 h at 100°C.

#### HRO/Na-β

HRO supported on Na-β zeolite was prepared by simultaneous precipitation of HRO and its impregnation on Na-β zeolite. The 0.001 M of RuCl_3_ solution is mixed with the appropriate amount of aqueous CaCO_3_ solution and the desired amount of zeolite, with pH maintained at ~7–8. The resulting mixture was stirred up to 12 h at room temperature; the obtained precipitate was washed with water and dried for 3 h at 100°C.

### Procedure for Catalytic Hydrogenation of Sugars

The reactions were carried out in a stainless steel (SS-316) high-pressure 100-ml reactor (Amar Equipment PVT. LTD. India), equipped with an electrically heated jacket with a mechanical stirrer. The reactor was loaded with the catalyst and the substrate (sugars) dissolved in water, purged with N_2_ three times before pressurizing with a fixed amount of H_2_, and the reaction was carried out at desired temperatures and time duration. After completion of the reaction, the reactor was cooled to room temperature and the excess H_2_ was released. The catalyst was separated by simple centrifugation and used for the next cycle without any pretreatment.

### Product Analysis

The quantitative analysis of product mixture was done by using the Shimadzu Ultra-High Performance Liquid Chromatography (UHPLC) system equipped with low-temperature evaporative light scattering detector (ELSD-LTII) using a Supelcogel-610H column. The mobile phase was distilled H_2_O with a flow rate of 0.5 ml min^−1^, and the column oven was set at 40°C.

### Catalyst Characterization

PXRD measurement was carried out in a Philips X'Pert MPD system using Cu Kα radiation (λ = 1.5406 Å). The operating voltage and current were 40 kV and 30 mA, respectively. A step size of 0.04° with a step time of 2 s was used for data collection. The data were processed using the Philips X'Pert (version 2.2e) software. Identification of the crystalline phases was made by comparison with the JCPDS files.

Thermogravimetric analysis (TGA) was carried out in Mettler-Toledo (TGA/SDTA 851^e^) and the data were processed using Star^e^ software, in air at a flow rate of 60 ml/min and at a heating rate of 10°C/min in the temperature range 50–900°C.

Transmission electron microscope (TEM) images were obtained with a JEOL JEM-2100 microscope with an acceleration voltage of 200 kV using carbon-coated 200 mesh copper/gold grids. The samples were ultrasonically dispersed in ethanol for 5 min and deposited onto carbon film using capillary and dried in air for 30 min.

The surface morphology studies were done with a scanning electron microscope (JEOL series JSM-7100F) equipped with Oxford instruments energy-dispersive X-ray spectrometer (EDX) facility. The samples were coated with gold using sputter coating before analysis to avoid charging effects during recording. Analyses were carried out with an accelerating voltage of 15 kV and a working distance of 10 mm, with magnification values in-between 500 × and 15,000 ×.

The acidity of HRO/Na-β was analyzed through pyridine adsorption and monitored using Fourier-transformed infrared (py-FTIR) spectroscopic technique. For py-FTIR analysis, the sample was initially oven-dried at 100°C for 3 h. To the oven-dried sample (50 mg), 0.1 ml of pyridine was admixed directly. The physisorbed pyridine present in the sample was dried in the oven at 120°C for 1 h to remove it. Further, the sample is cooled to room temperature, the spectra were recorded with a nominal resolution of 4 cm^−1^ in the spectral range of 400–4,000 cm^−1^ using a KBr background, and 15 scans were accumulated for spectrum.

Elemental chemical analyses of the samples were determined using inductively coupled plasma emission spectrometry (ICPOES; Perkin Elmer, OES, Optical 2000 DV). The samples were digested in a minimum amount of concentrated HNO_3_ and H_2_SO_4_ further diluted using milli Q water <10 ppm and analyzed.

Specific surface area and pore size analysis of the samples were measured by nitrogen adsorption at −196°C using a sorptometer (ASAP-2020, Micromeritics). The samples were degassed under vacuum at 80°C for 90 min prior to measurements in order to expel the interlayer water molecules. The BET-specific surface area was calculated by using the standard Brunauer, Emmett, and Teller method on the basis of adsorption data.

## Results and Discussions

### Studies of Catalyst Screening and Reaction Optimization Parameters for Hydrogenation of Xylose to Xylitol

HRO pre-catalyst was synthesized to demonstrate selective hydrogenation of sugars (xylose, glucose, and mannose) to sugar alcohols (xylitol, sorbitol, and mannitol) catalyzed by *in situ* generated Ru(0) active species. An initial blank experiment on xylose hydrogenation was conducted at 120°C, 30 bar H_2_ for 1 h, in absence of catalyst; no reaction was noted (Table 1, entry 1). During catalyst screening, 5% Ru/C showed 100% conversion of xylose with 99% yield of xylitol (Table 1, entry 2). Then, we checked the catalytic efficiency of our Na-β zeolite supported HRO material (HRO/Na-β) and it showed conversion and yield similar to 5% Ru/C catalyst (Table 1, entry 3). The energy-efficient preparation of *in situ* generated Ru-HRO@Na-β catalyst (explained in the catalyst characterization) from HRO/Na-β during reaction was interesting to us. Hence, we have explored this material for the hydrogenation of various sugars to sugar alcohols. In all the hydrogenation reactions, we have observed 100% selectivity of the desired sugar alcohols, and thus only the conversions (%) of reactants (sugars) are mentioned in the subsequent sections.

**Table 1:** Catalyst screening and optimization reaction condition for the hydrogenation of xylose to xylitol^a^.

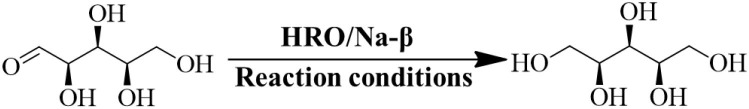						
**Entry**	**Catalyst/Pre-catalyst**	**Catalyst/Pre-catalyst (mg)**	**Temp (°C)**	**Pressure in bar (H**_**2**_**)**	**Time (min)**	**Conv. (%)**
1	Blank	-	120	30	60	n.o
2	5% Ru/C	50	120	30	60	99
3	HRO/Na-β	50	120	30	60	100
4	HRO/Na-β	50	100	30	60	100
5	HRO/Na-β	50	80	30	60	100
6	HRO/Na-β	50	60	30	60	80
7	HRO/Na-β	50	80	20	60	100
8	HRO/Na-β	50	80	10	60	60
9	HRO/Na-β	50	80	20	30	100
10	HRO	2.5	80	20	30	85
11	RuO_2_	2.5	80	20	30	30
12^b^	HRO	2.5	80	20	30	8
13	Ru-HRO-1	2.5	80	20	30	84
14^c^	HRO/Na-β	250	80	50	100	>99
15^d^	HRO/Na-β	500	80	50	68	99
16^e^	HRO/Na-β	1000 (1 g)	80	50	47	99
17^e^	Ru-HRO@Na-β^f^	950	80	50	26	99

a Reaction conditions: 1 g of xylose in 40 ml of H_2_O, 50 mg of HRO/Na-β pre-catalyst (5 wt% of Ru), 60–120°C, 10–30 bar H_2_, 30–60 min.

b 1 g of xylose in 40 ml of methanol.

c 5 g of xylose in 40 ml of H_2_O.

d 10 g of xylose in 40 ml of H_2_O.

e 15 g of xylose in 40 ml of H_2_O.

f *Recovered catalyst from entry 16; n.o, Not observed*.

Reaction parameters such as temperature, hydrogen pressure, and reaction time were varied using HRO/Na-β as the catalyst precursor in order to identify a mild reaction condition for the hydrogenation of sugars. Xylose hydrogenation is studied for optimization of reaction conditions. The temperature was decreased from 120 to 60°C by steps of 20°C at 30 bar H_2_ for 1 h using 5 wt% of HRO/Na-β (50 mg) (Table 1, entries 3–6). Complete conversion of xylose was observed at 120, 100, and 80°C, and a decreased conversion to 80% was observed on further reducing the temperature to 60°C. Thus, the temperature was fixed at 80°C for subsequent reactions. H_2_ pressure was varied from 30 to 10 bar by a factor of 10 at 80°C for 1 h, and it was observed that 20 bar H_2_ was sufficient for the complete conversion of xylose. Ten bar H_2_ showed a decrease in conversion of xylose to 77%; thus, the H_2_ pressure was fixed at 20 bar (Table 1, entries 5, 7, and 8). After having optimized the temperature (80°C) and pressure (20 bar H_2_), the reaction time was decreased from 60 to 30 min. Complete conversion of xylose to xylitol was observed even after 30 min of reaction (Table 1, entry 7 and 9).

**Figure 1:**
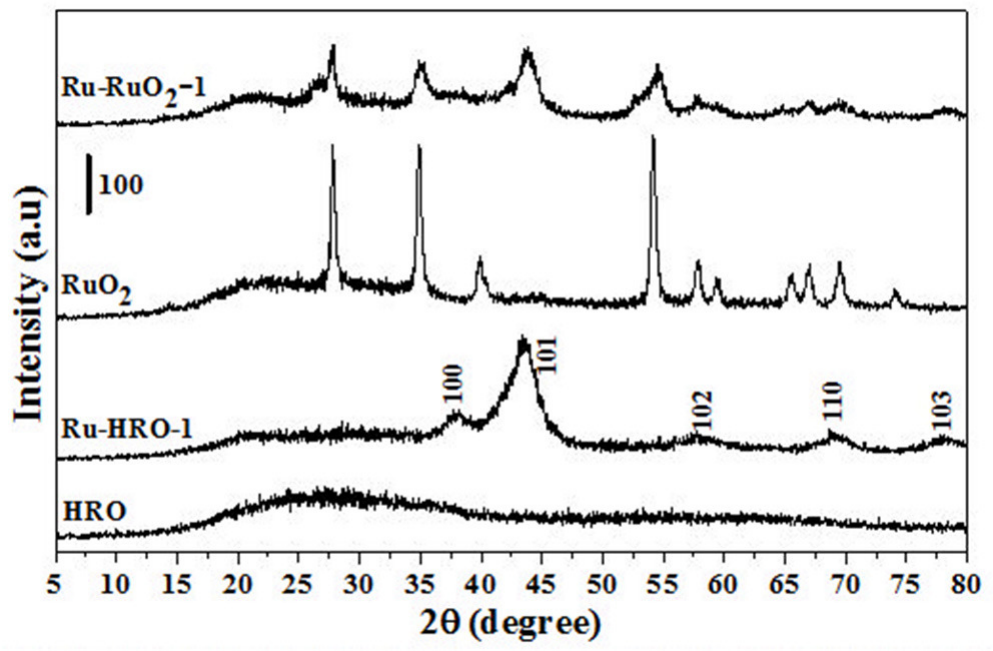
PXRD of materials before (HRO and RuO_2_) and after reaction (Ru-HRO-1: Recovered material from Table 1, entry 10 and Ru-RuO_2_-1: Recovered material from Table 1, entry 11).

The xylose hydrogenation was also conducted with un-supported HRO at optimized reaction conditions. After the reaction, the obtained material, named Ru-HRO-1 [HRO is not completely converted to Ru(0)], showed 85% conversion of xylose (Table 1, entry 10). The supported HRO on Na-β-zeolite (HRO/Na-β) showed 100% conversion, implying that the support is playing a positive role to improve the catalytic activity of Ru-HRO-1. Ru(0) sites are well-dispersed in the support and hence easily accessible to the substrate molecules. This was confirmed from TEM analysis (Figure 8D). In case of un-supported Ru-HRO, Ru particles are agglomerated (Figure 8B), effectively reducing the available active sites and hence conversion observed in this case was less when compared with supported Ru-HRO. In addition, the supported catalyst is easy to remove after the reaction as compared to Ru-HRO-1. Xylose hydrogenation was also conducted with RuO_2_ and resulted in a decrease in conversion (30%) as compared with the HRO material (Table 1, entries 10 and 11). Under the reaction conditions, the RuO_2_ generates a smaller number of Ru(0) active catalytic species compared to HRO, which is demonstrated by PXRD (Figure 1). The PXRD of Ru-HRO-1 (recovered material from Table 1, entry 10) showed a high-intensity peak of Ru(0) at 43°, which was less in Ru-RuO_2_-1 (recovered material from Table 1, entry 11). TPR analysis revealed that HRO reduction started at a lower temperature (135°C) compared to RuO_2_ (250°C) (Gundekari and Srinivasan, 2019). Using HRO, reaction conducted in the presence of methanol as solvent showed only 8% conversion of xylose with 100% selectivity for xylitol (Table 1, entry 12). Compared to aqueous medium, the organic solvents proved to be less effective for the conversion of HRO to active Ru(0). The detailed explanation of the effect of solvents in the conversion HRO to Ru (0) is mentioned in section Catalyst Characterization. The catalytic activity was successfully demonstrated at a 5-g scale of xylose, and >99% conversion of xylose was achieved without compromising the selectivity of xylitol under the optimized reaction conditions (Table 1, entry 14). The concentration of xylitol was further increased to 10 g (25 wt%) and 15 g (37.5 wt%) scale, and a decrease in the conversion [i.e., 68 and 47% (Table 1, entry 15 and 16), was observed]. The decrease in the catalytic activity is due to the decrease in the Ru wt% in the catalyst. The product mixture in the case of the 10- and 15-g scale turned light green in color from a colorless solution, which is presumably due to the leaching of Ru metal from zeolitic support.

### Optimization of Reaction Conditions for Hydrogenation of C_6_-Sugars (Glucose and Mannose) to Sugar Alcohols (Sorbitol and Mannitol) Using HRO/Na-β Pre-catalyst

The optimized reaction condition for the conversion of xylose to xylitol is 80°C, 20 bar H_2_ for 30 min. Hydrogenation of glucose was attempted under this reaction condition; 85% conversion was observed for glucose (Table 2, entry 1). The decrease in conversion may be attributed to the difference in the size of the molecule from xylose to glucose. Actually, the Ru particles formed via *in situ* reduction of HRO are very small (average particle size ~ 1–2 nm; Figure 8). Some of the finer Ru particles deposit on the pores (0.67 nm) of β-zeolite support (Hao et al., 2018). The Stokes diameter of xylose is 0.64 nm, which can easily enter β-zeolite pores and interact with the ultrafine Ru particles. Owing to the highly active nature of these Ru particles, the hydrogenation of xylose was accelerated (Sjoman et al., 2007; Roli et al., 2016). The Stokes diameter of glucose is 0.73 nm; it cannot go inside the pores of β-zeolite and does not interact with such ultrafine Ru particles, which may be the reason for the activity difference in xylose and glucose (Sjoman et al., 2007; Roli et al., 2016). To improve the conversion of glucose, the reaction parameters were modified (temperature increased from 80 to 100°C and reaction time enhanced from 30 min to 45 min). Under these reaction conditions (100°C, 20 bar H_2_ for 45 min), glucose was completely converted to sorbitol (Table 2, entries 2 and 3). Similar conversion and selectivity were also observed for mannose hydrogenation to mannitol (Table 2, entry 4).

**Table 2:** Optimization of reaction condition for glucose and mannose hydrogenation to sorbitol and mannitol using HRO/Na-β pre-catalyst^a^.

**S. No**	**Temp. (^**°**^C)**	**H_**2**_ pressure in bar**	**Time (min)**	**Conv. (%)**
1^b^	80	20	30	85
2^b^	100	20	30	92
3^b^	100	20	45	100
4^c^	100	20	45	100

a Reaction conditions: 1 g of carbohydrate in 40 ml of H_2_O, 50 mg of HRO/Na-β pre-catalyst (5 wt%).

b Glucose.

c *Mannose*.

The catalytic activity is dependent on the reaction temperature and pressure (H_2_). An increase in these parameters increases the reduction capacity of HRO due to an increased conversion to Ru(0), which is the active species for hydrogenation. Increasing reaction temperature from 100 to 200°C and pressure (H_2_) from 20 to 40 bar completed the conversion of glucose to sorbitol within 10 min of time (Supplementary Table 3, entries 1–3). Increase in the reduction of HRO on changing the parameters was supported from various physicochemical techniques, and discussed in the catalyst characterization section.

### Recyclability of Ru-HRO@Na-β Catalyst for Xylose Conversion to Xylitol

The recyclability of a catalyst is attractive for bulk chemical synthetic industrial applications. After the xylose-to-xylitol conversion (Supplementary Table 2, entry 1), the catalyst was removed from the product mixture by simple centrifugation, washed with deionized water, and further used for the next cycle under our optimized reaction conditions. The observed catalytic activity was comparable to the fresh catalyst (Supplementary Table 2, entry 2). The same procedure was followed for four more reaction cycles, and similar catalytic activity [i.e., 98–99% conversion of xylose with 100% selectivity (Supplementary Table 2, entries 3–6), was observed]. The ICP analysis showed the leaching of a negligible amount of Ru metal into the aqueous product mixture after these reaction cycles. At a higher concentration of the sugar solution, the recyclability of the catalyst is poor because of significant amount of Ru metal leaching from the support. We observed only 26% of conversion in the second cycle of the 15-g scale where the conversion was 47% in the fresh cycle (Table 1, entries 16 and 17).

### Catalyst Characterization

**Figure 2:**
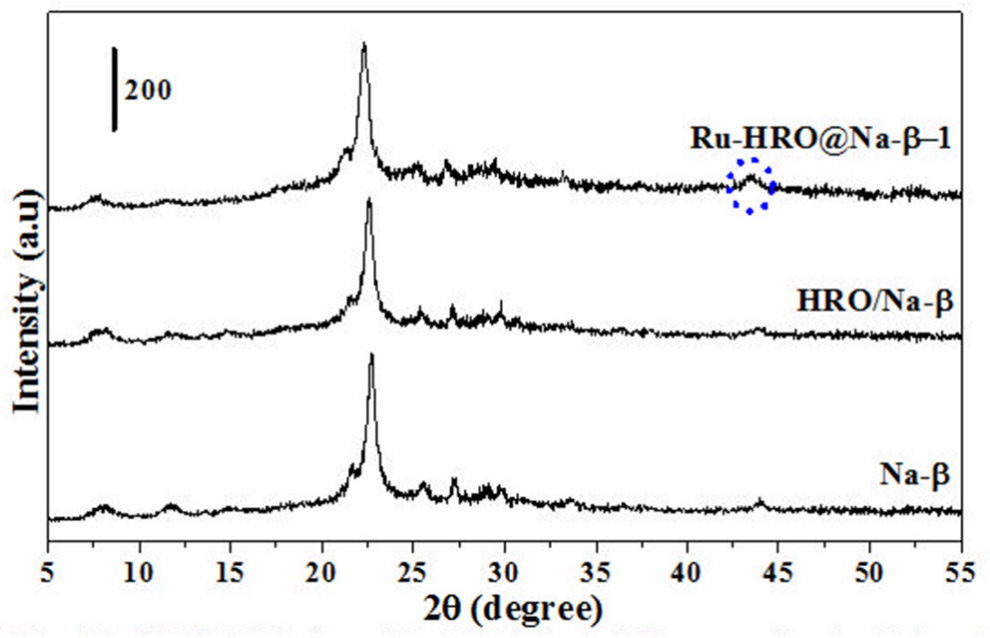
PXRD of Na-β, HRO/Na-β, and Ru-HRO/Na-β-1 (Recovered material from Supplementary Table 2, entry 1).

HRO consists of Ru in multiple oxidation states, and the material acts as a pre-catalyst (catalyst precursor). Under hydrogen environment at elevated temperatures, HRO *in situ* generates Ru(0) nanoparticles, which is the active catalytic species. *In situ* formation of Ru(0) from HRO is studied by various analytical tools. The PXRD of HRO did not show any diffraction peaks attesting to its amorphous nature. After the reaction (Table 1, entry 10), the obtained material (Ru-HRO-1) showed diffraction peaks at 2θ of 38.3, 41.9, 43.7, 58.3, 69.4, and 78.4, corresponding to (100), (002), (101), (102), (110), and (103) planes of the hexagonal close-packed (hcp) Ru metal, respectively (ICDD-JCPDS card No. 06-0663) (Figure 1). The Na-β support shows diffraction peaks at 7.2, 21.4, 22.4, 25.2, 27.0, 28.7, and 29.5, which were characteristic peaks of this zeolite. Similar peaks were observed after impregnation of HRO, which indicate that the HRO impregnation did not affect the crystallinity of the zeolite. Na-β zeolite support retained its crystallinity even after the reaction. HRO was converted to Ru(0), as was depicted from the new peak at 43°, corresponding to Ru(0) (Figure 2).

**Figure 3:**
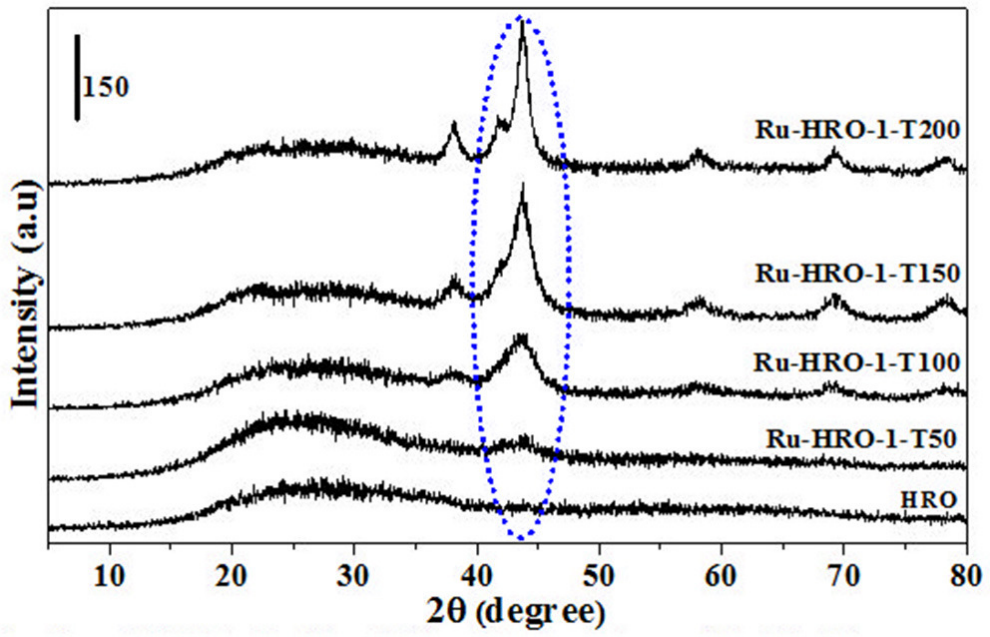
Reduction of HRO to Ru(0) at different temparatures (50–200°C). Reaction conditions: 25 mg of HRO in 40 mL of H_2_O, 20 bar H_2_, 10 min.

**Figure 4:**
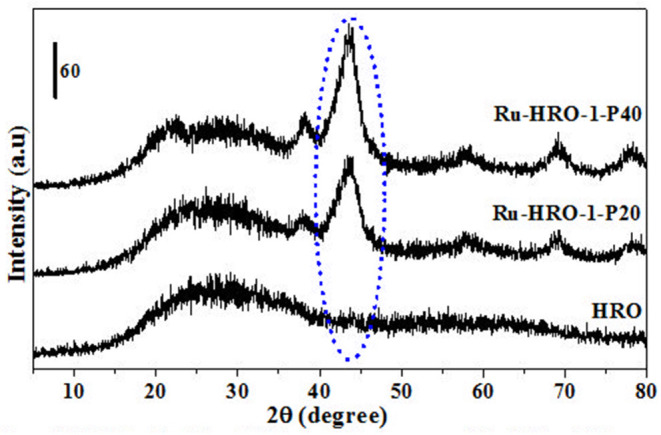
Reduction of HRO to Ru(0) at different H_2_ pressure (20–40 bar H_2_). Reaction conditions: 25 mg of HRO in 40 mL of H_2_O, 100°C, 10 min.

A set of reactions were conducted for 10 min in aqueous medium at different temperatures to understand the reduction of HRO (Figures 3, 4). The reduction of HRO to Ru(0) critically depends on the temperature and H_2_ pressure. The reduction is dependent on the temperature and H_2_ pressure; increasing these parameters increased the reduction efficiency. The PXRD profile of the obtained Ru-HRO-1 material from HRO at different temperatures (50, 100, 150, and 200°C) was monitored in Figure 3 (the materials denoted as Ru-HRO-1-T50, Ru-HRO-1-T100, Ru-HRO-1-T150, and Ru-HRO-1-T200) which reveals an increase in the intensity of Ru(0) peak with the increase in reaction temperature. When the H_2_ pressure was increased from 20 to 40 bar, we observed a similar increase in the intensity of Ru(0) peak (Figure 4) and materials are denoted as Ru-HRO-1-P20 and Ru-HRO-1-P40.

**Figure 5:**
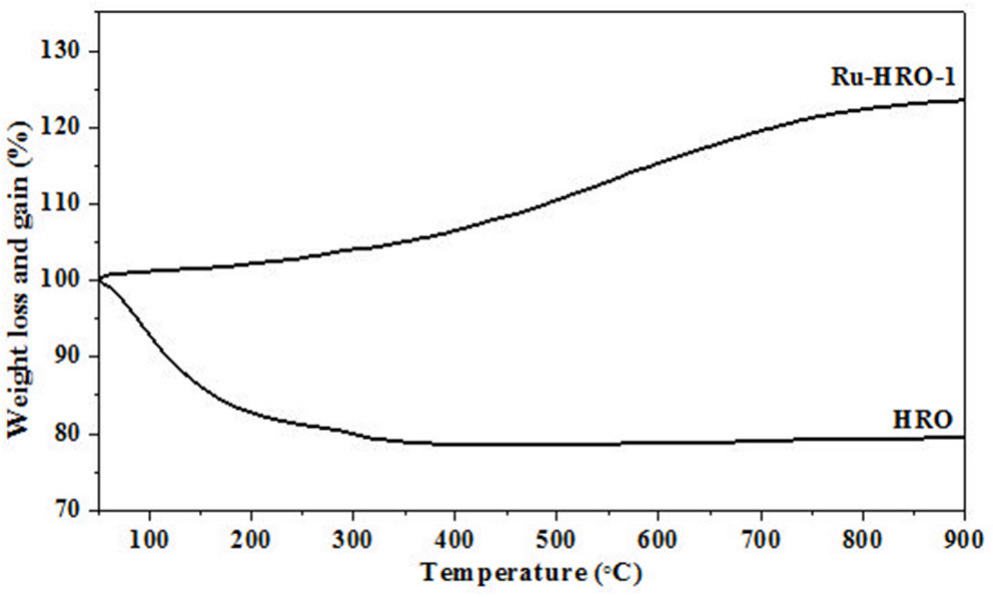
TGA of HRO and Ru-HRO-1 (Recovered material from Supplementary Table 3, entry 2).

**Figure 6:**
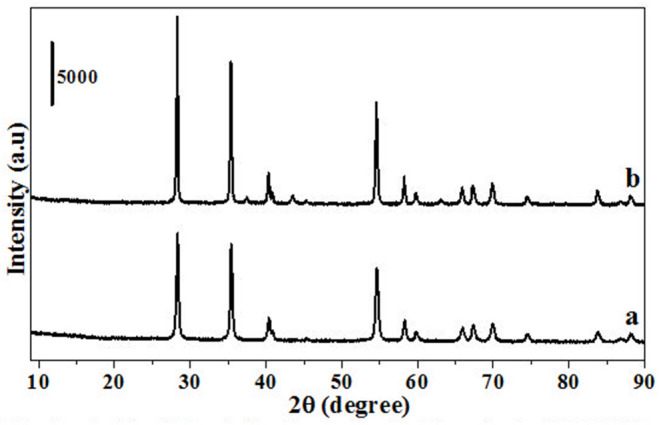
PXRD of materials obtained after thermogravimetric analysis. **(a)** HRO **(b)** Ru-HRO-1.

TGA of materials are shown in Figure 5. The weight loss of HRO (22%) was observed in the temperature range of 50 to 350°C. The weight loss at the temperature range 50 to <200°C is consistent with the loss of physisorbed H_2_O molecules on the HRO and >200 to 350°C temperature range is attributed to strongly held H_2_O molecules in HRO. After TGA, the obtained material is crystalline RuO_2_, which is confirmed by PXRD, due to the presence of intense peaks corresponding to 110, 101, 200, 211, 220, 002, 310, 112, 301, and 201 planes of tetragonal RuO_2_ (JCPDS card no. 21-1172). This implies that the presence of strongly held H_2_O molecules results in the amorphous nature of HRO (Figure 6a). On the other hand, at 200°C (Figure 3), Ru(0) was generated from HRO during the reaction. The material obtained after the reaction showed a weight gain (23%) in TGA gradually from 50 to 900°C. In the presence of air atmosphere, the Ru(0) particles generated in the course of the reaction forms the RuO_2_ under TGA conditions, and it was the reason for the observed weight gain (Figure 6b). The experiment also supported the *in situ* formation of Ru(0) from HRO during the reaction.

**Figure 7:**
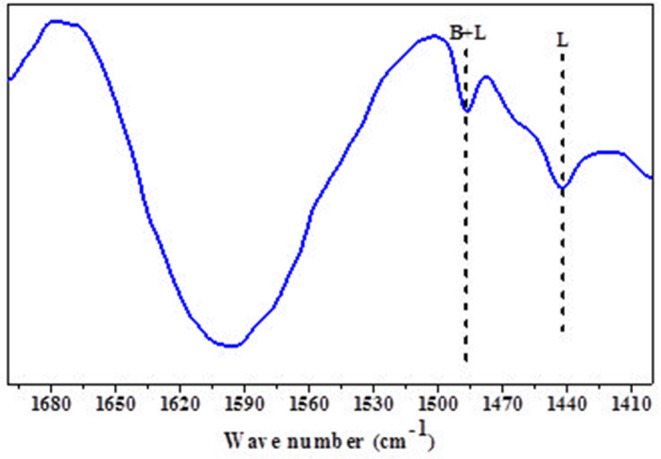
py-FTIR of the catalyst precursor HRO/Na-β.

Mishra et al. disclosed that mild acidity of support (zeolites) increases the selectivity of sugar hydrogenated product (Mishra et al., 2014). The py-FTIR of the catalyst precursor (HRO/Na-β) shows a peak between 1,490 and 1,480 cm^−1^, corresponding to a mixture of Brönsted and Lewis acidic sites. Another peak observed at 1,450–1,435 cm^−1^ identifies the Lewis acidic sites (Figure 7).

**Figure 8:**
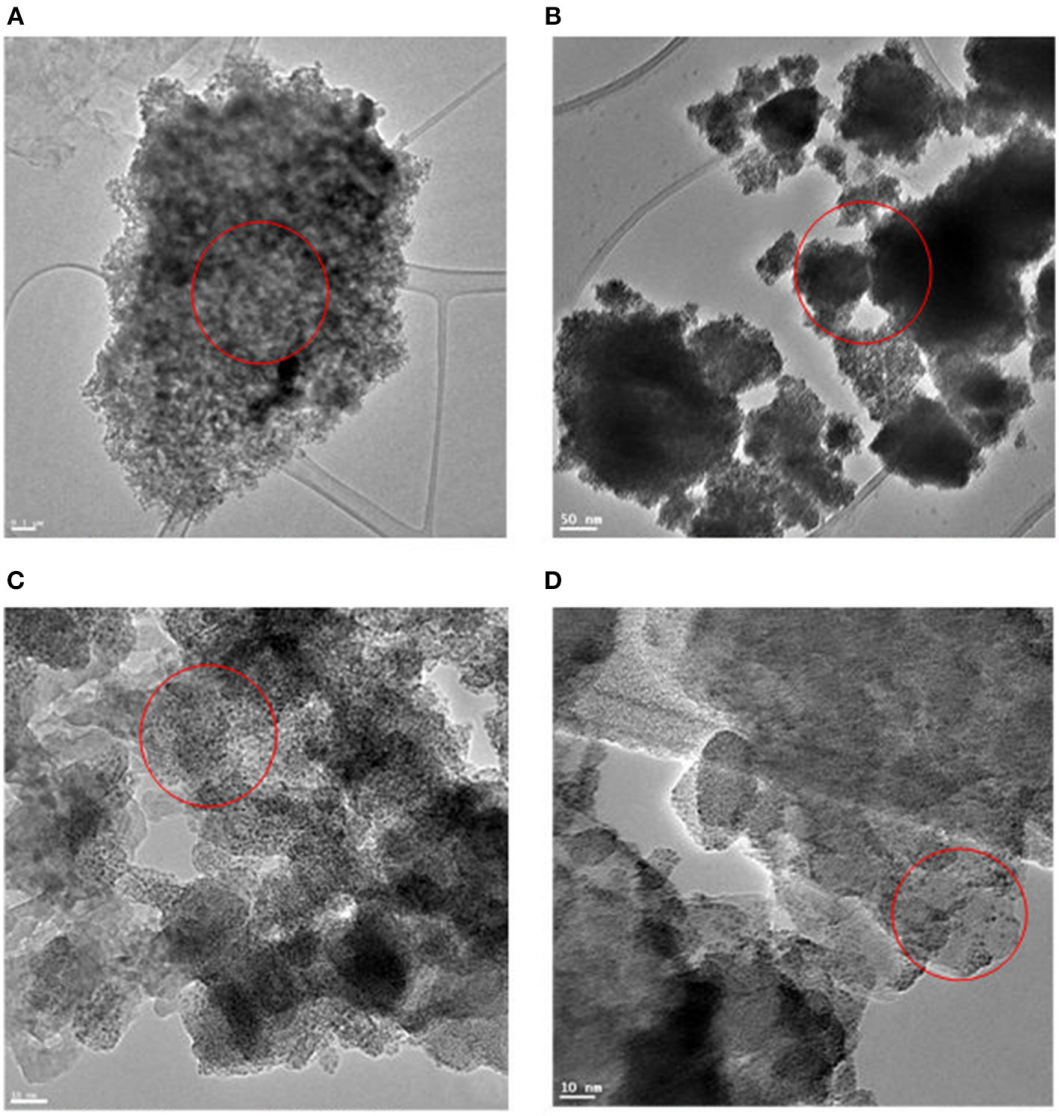
TEM image of materials. **(A)** TEM image of HRO. **(B)** TEM image of Ru-HRO-2 (Recovered material from Table 1, entry 13). **(C)** TEM image of HRO/Na-β. **(D)** TEM image of *in situ* formed Ru-HRO@Na-β-2 (Recovered material from Supplementary Table 2, entry 2).

**Figure 9:**
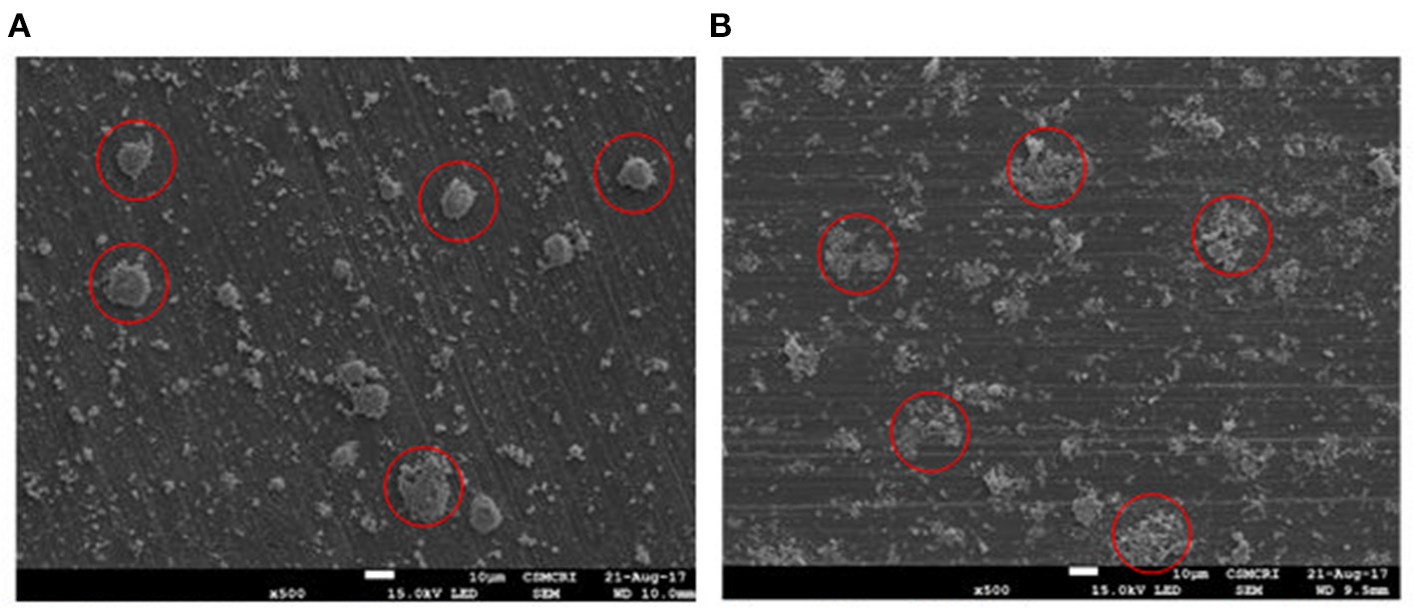
SEM image of materials. **(A)** SEM image of HRO. **(B)** SEM image of Ru-HRO-2 (Recovered material from Table 1, entry 13).

The TEM images of Ru in HRO particles showed agglomeration. After the reaction, the obtained material (here, Ru-HRO-2; recovered material from Table 1, entry 13) showed a decrease in agglomeration, which might be due to a cleavage of Ru-O-Ru linkages while forming Ru(0) from HRO (Figures 8A,B). A similar observation was found in SEM analysis also; HRO consists of bulk clusters on the grid, but in the case of Ru-HRO-2 (recovered from Table 1, entry 13), a decrease in the clusters concomitant to an increase in individual particles were observed (Figures 9A,B). SEM-EDX is shown in Supplementary Figures 2A,B. HRO consists of 50% of oxygen and the remaining is Ru. A decrease in the oxygen content was observed for Ru-HRO-2 (recovered material from Table 1, entry 13) up to 29%. The oxygen and water molecules in HRO were removed at elevated temperatures under reductive environment of our reaction conditions resulting in Ru(0). It was also confirmed that the entire HRO is not reduced in a single reaction cycle and the Ru(0) amount increases with successive reaction cycles. The TEM images of HRO supported on Na-β showed that the HRO clusters were well-dispersed on the support and the Ru-HRO@Na-β-2 (recovered material from Supplementary Table 2, entry 2) showed some divided particles ascribed to Ru(0) along with some clusters attributed to the un-converted HRO (Figures 8C,D).

**Figure 10:**
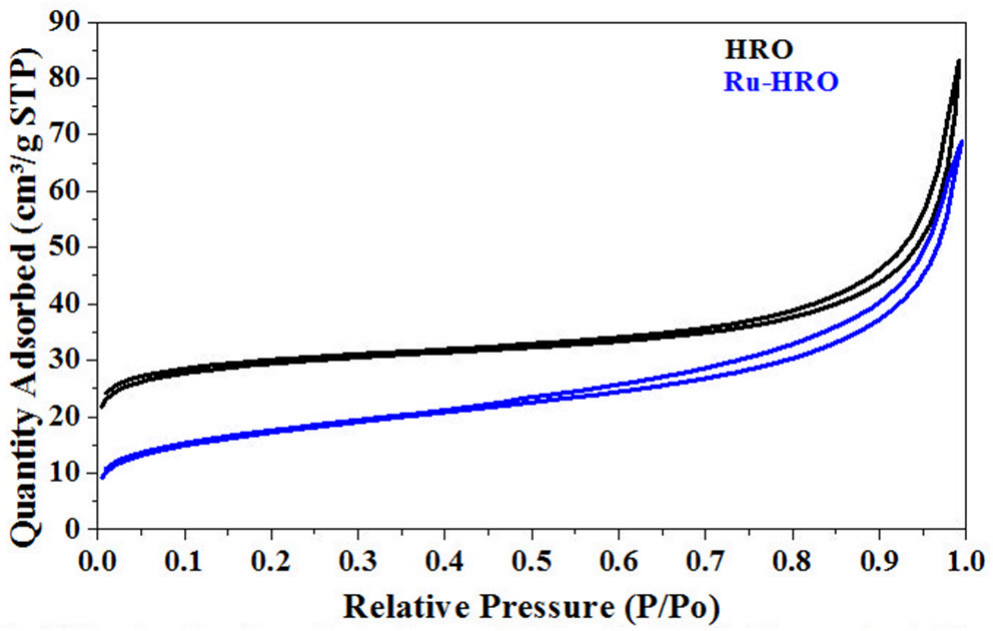
N_2_ absorption-desorption isotherms of HRO and Ru-HRO-1 (Recovered material from Table 1, entry 10).

The BET-specific surface area of the HRO was measured as 94 m^2^/g. The nitrogen adsorption isotherms for HRO showed a characteristic type-IV isotherm that was attributed to the capillary condensation of pores with H1-type hysteresis according to IUPAC classification (Figure 10) (Luxton et al., 2011). After the reaction (Table 1, entry 10), the obtained material/*in situ* catalyst Ru-HRO-1 showed 60 m^2^/g and retained type-IV isotherm with H1-type hysteresis. We presume that the surface area comes from the existence of HRO in the material. Under the reaction conditions, some of the HRO converts to Ru(0) and thereby reduces the amount of HRO, causing a decrease in the surface area. The surface area and type-IV isotherm observed after the reaction was due to the unconverted HRO in the Ru-HRO-1 catalyst (Figure 10). According to our previous work, HRO has Ru in +6 and +3 oxidation states. The *in situ* generated Ru-HRO catalyst exhibited (0) and +4 oxidation states, which means during the reaction under H_2_, the +6 is transformed to +4 and +4 is transformed to (0) (Gundekari and Srinivasan, 2019).

**Figure 11:**
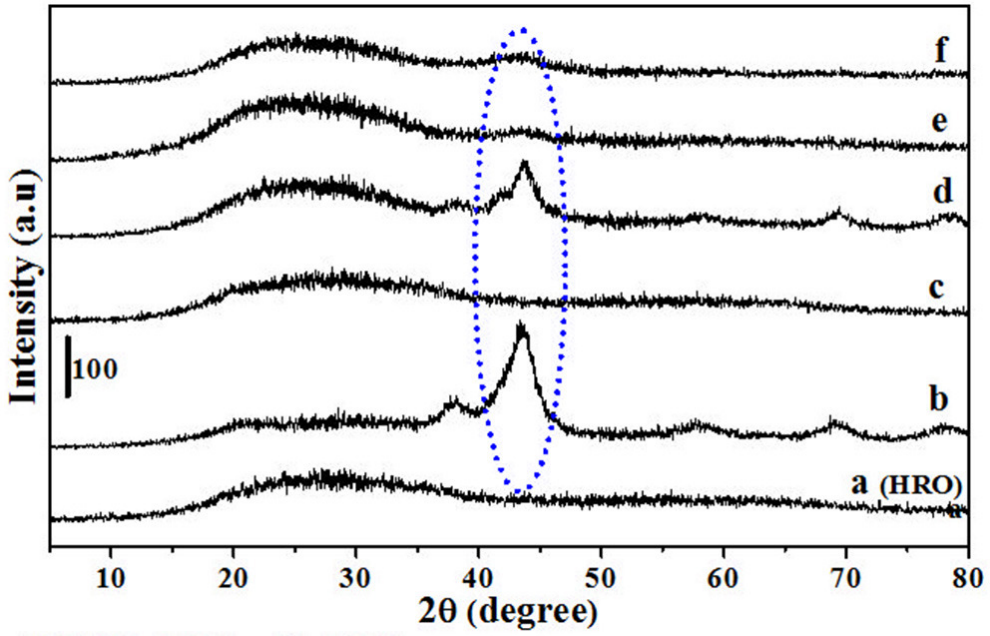
PXRD of **(a)** HRO Ru-HRO-1. The denotation of **(b–f)** mentioned in Table 3.

The reduction behavior of HRO was studied by conducting a set of reactions mentioned in Table 3 and materials obtained after the reaction were characterized using PXRD (Figure 11a belongs to HRO). The reduction of HRO to Ru(0) was observed in the presence of H_2_O in the medium and H_2_ environment (Table 3, entry 1; Figure 11b). Aqueous medium in the absence of H_2_ did not promote HRO reduction, indicating that H_2_O was not participating the reduction of HRO. Thus, the H_2_ consumed for this conversion (HRO to Ru-HRO) is obtained from the molecular H_2_ only (Table 3, entry 2; Figure 11c). The reaction conducted with only H_2_ in the absence of H_2_O and/or any medium showed less Ru(0) in the obtained material as compared with Table 3, entry 1. This result indicates that water facilitates the reaction by increasing the availability of H_2_ to HRO (Table 3, entry 3; Figure 11d). In the presence of H_2_, other solvents such as methanol and tetrahydrofuran (THF) showed much less conversion of HRO to Ru(0) (Table 3, entries 4 and 5; Figures 11e,f) as compared with aqueous medium and solvent-free conditions (Table 3, entry 3). From the above set of experiments, the suitability of water as the reaction medium for conducting hydrogenation reactions in the presence of HRO as the pre-catalyst was ascertained.

**Table 3:** Reduction behavior of HRO.

**Entry**	**H_**2**_ source**	**Solvent**	**PXRD**
1	H_2_	H_2_O	Figure 11b
2	-	H_2_O	Figure 11c
3	H_2_	-	Figure 11c
4	H_2_	THF	Figure 11e
5	H_2_	Methanol	Figure 11f

*Reaction conditions: 25 mg of HRO material, 40 ml of solvent, 100°C, 20 bar H_2_, 30 min*.

### Reaction Mechanism

The proposed mechanism of reduction of sugar alcohols is depicted in Supplementary Figure 3 considering the reaction conditions employed. Initially, under a hydrogen environment, a certain amount of HRO is converted to Ru(0), which is the active species for the hydrogenation. The hydrogen molecules are adsorbed on the surface of the *in situ* generated Ru(0) and form metal–hydrogen bonds. The sugar molecules adsorbed on the surface and in close proximity of Ru-H bonds undergo hydrogenation of the carbonyl group of the sugar molecule (HC=O) to alcohol, thereby forming the corresponding sugar alcohol (CH-OH). The alcohols once formed are desorbed from the surface. Subsequently, Ru(0) metal (freshly formed from HRO or used species for hydrogenation) interacts with the available hydrogen and the reaction continues thereafter as indicated above until the complete conversion of sugar molecules to sugar alcohols.

## Conclusion

We have successfully demonstrated the selective hydrogenation of sugars (xylose, glucose, and mannose) to corresponding sugar alcohols (xylitol, sorbitol, and mannitol) with 100% yields using HRO/Na-β pre-catalyst under optimized reaction conditions (80–100°C, 20 bar H_2_, 30–45 min). *In situ* formation of Ru(0) from HRO during the reaction is characterized by several physico-chemical techniques. Control experiments support the idea that the reduction reaction under aqueous condition is efficient and beneficial for the conversion of HRO to Ru(0) as compared to organic solvents such as methanol and THF. The present catalytic method has the advantage of minimizing the energy and H_2_ consumption of the overall process by avoiding external reduction (generally used in the conventional catalytic processes). Moreover, the *in situ* generation of catalyst precludes the need for co-catalysts and additives and has good recyclability.

## Data Availability Statement

All datasets generated for this study are included in the article/Supplementary Material.

## Author Contributions

SG: designed and worked for the manuscript. HD, KR, and JM: helped in optimization and characterization studies and proof-editing of the manuscript. KS: overall supervision of the work and in writing the manuscript. All authors contributed to the article and approved the submitted version.

## Conflict of Interest

The authors declare that the research was conducted in the absence of any commercial or financial relationships that could be construed as a potential conflict of interest.

## Abbreviations

HRO: Hydrous ruthenium oxide
Ru-HRO: *In situ* generated Ru along with HRO (general denotation)
Ru-HRO-1: *In situ* generated Ru along with HRO (after 1st cycle using HRO catalyst precursor)
Ru-HRO-2: *In situ* generated Ru along with HRO (after 2nd cycle using Ru-HRO-1)
HRO/Na-β: HRO supported Na-β (catalyst precursor)
Ru-HRO@Na-β: *In situ* generated Ru along with HRO on Na-β (general denotation)
Ru-HRO@Na-β-1: *In situ* generated Ru along with HRO on Na-β (after 1st cycle using HRO/Na-β catalyst precursor)
Ru-HRO@Na-β-2: *In situ* generated Ru along with HRO on Na-β (after 2nd cycle using Ru-HRO@Na-β-1)
Ru-RuO_2_-1: *In situ* generated Ru along with RuO_2_ (after 1st cycle using RuO_2_ catalyst precursor).
