# Supplementary material for: In situ Generated Ru(0)-HRO@Na-β From Hydrous Ruthenium Oxide (HRO)/Na-β: An Energy-Efficient Catalyst for Selective Hydrogenation of Sugars
Source: Front Chem. 2020 Nov 25;8:525277. doi: 10.3389/fchem.2020.525277 (PMC7724086; doi:10.3389/fchem.2020.525277)
Supplement: Supplementary file 1 [file Data_Sheet_1.DOCX]

***In situ* generated Ru(0)-HRO@Na-β from Hydrous Ruthenium Oxide (HRO)/Na-β: An energy efficient catalyst for selective hydrogenation of sugars**

**Sreedhar Gundekari,^a,b^ Heena Desai,^a^ Krishnan Ravi,^a,b^ Joyee Mitra,^a,b^ and Kannan Srinivasan^a,b^***

*^a^Inorganic Materials and Catalysis Division, CSIR-Central Salt and Marine Chemicals Research Institute, Council of Scientific and Industrial Research (CSIR), GB Marg, Bhavnagar* - *364 002, India*

*^b^Academy of Scientific and Innovative Research, CSIR-Central Salt and Marine Chemicals Research Institute, GB Marg, Bhavnagar-364 002, India*

| Table S1 Comparison of HRO/Na-β pre-catalyst with reported catalysts for hydrogenation of sugars to sugar alcohols | | | | | | | |
| --- | --- | --- | --- | --- | --- | --- | --- |
| **S.No** | **Reactant** | **Catalyst** | **Reaction condition** | **External reduction** | **Con. (%)** | **Sel. (%)** | **Ref.** |
| 1 | Xylose | Ru/HYZ | 120 ℃, 55 bar H_2_, 2 h | NaBH_4_ (RT in ethanol) | 99 | 98 | 1 |
| 3 | Xylose | Ru/TiO_2_ | 120 ℃, 20 bar H_2_, 15 min | 350 ℃, 2 h Under H_2_ | 100 | 95 | 2 |
| 2 | Glucose | Ru/MCM-41 | 120 ℃, 30 bar H_2_, 2 h | HCHO (RT) | 100 | 98 | 3 |
| 4 | xylose | Perovskite Ni | 100 ℃, 25 bar H_2_, 5 h | 500 ℃, 2 h Under H_2_ | 100 | 51 | 4 |
| 5 | Mannose | Ru/NiO-TiO_2_ | 120 ℃, 40-55 bar H_2_, 4 h | (5.0%) H_2_/Ar at  200 ℃ for 3 h | 94 | 91 | 5 |
| 6 | Glucose | Ru/ZSM‐5 | 120 ℃, 40 bar H_2_, 2 h | 350 ℃, 3 h Under H_2_ | 99 | 97 | 6 |
| 7 | Glucose | NiNPs/AlOH | 120 ℃, 40 bar H_2_, 24 h | - | 99 | 96 | 7 |
| 8 | Mannose | NiNPs/AlOH | 120 ℃, 40 bar H_2_, 24 h | - | 99 | 96 | 7 |
| 9 | Xylose | HRO/Na-β pre-catalyst | 80 °C, 20 bar H_2_, 30 min | - | 100 | 100 | Present work |
| 10 | Glucose |  | 100 °C, 20 bar H_2_, 45 min | - | 100 | 100 |  |
| 11 | Mannose |  | 100 °C, 20 bar H_2_, 45 min | - | 100 | 100 |  |

**Reference:**

1. Mishra, D.K., Dabbawala, A.A., and Hwang, J.-S. (2013). Ruthenium nanoparticles supported on zeolite Y as an efficient catalyst for selective hydrogenation of xylose to xylitol. J. Mol. Catal.A Chemical. 376, 63-70. doi: 10.1016/j.molcata.2013.04.011.
2. Hernandez-Mejia, C., Gnanakumar, E.S., Olivos-Suarez, A., Gascon, J., Greer, H.F., Zhou, W., Rothenberg, G., and Raveendran Shiju, N. (2016). Ru/TiO_2_-catalysed hydrogenation of xylose: the role of the crystal structure of the support. Catal. Sci. Technol. 6, 577-582. doi: 10.1039/C5CY01005E.
3. Zhang, J., Lin, L., Zhang, J., and Shi, J. (2011). Efficient conversion of D-glucose into D-sorbitol over MCM-41 supported Ru catalyst prepared by a formaldehyde reduction process. Carbohydr. Res. 346, 1327-1332. doi: 10.1016/j.carres.2011.04.037.
4. Morales, R., Campos, C.H., Fierro, J.L.G., Fraga, M.A., and Pecchi, G. (2016). Perovskite as nickel catalyst precursor - impact on catalyst stability on xylose aqueous-phase hydrogenation. RSC Adv. 6, 67817-67826. doi: 10.1039/C6RA13395A.
5. Mishra, D.K., and Hwang, J.-S. (2013). Selective hydrogenation of d-mannose to d-mannitol using NiO-modified TiO_2_ (NiO-TiO_2_) supported ruthenium catalyst. Appl. Catal. A: Gen. 453, 13-19. doi: 10.1016/j.apcata.2012.11.042.
6. Guo, X., Wang, X., Guan, J., Chen, X., Qin, Z., Mu, X., and Xian, M. (2014). Selective hydrogenation of D-glucose to D-sorbitol over Ru/ZSM-5 catalysts. Chinese J. Catal. 35, 733-740. doi: 10.1016/S1872-2067(14)60077-2.
7. Rodiansono, R., and Shogo, S., (2013). Effective Production of Sorbitol and Mannitol from Sugars Catalyzed by Ni Nanoparticles Supported on Aluminium Hydroxide. Bulletin of Chemical Reaction Engineering & Catalysis. 8, 40-46. doi: 10.9767/bcrec.8.1.4290.40-46.

|  |
| --- |
| Fig. S1 Applications of xylitol and sorbitol |

| 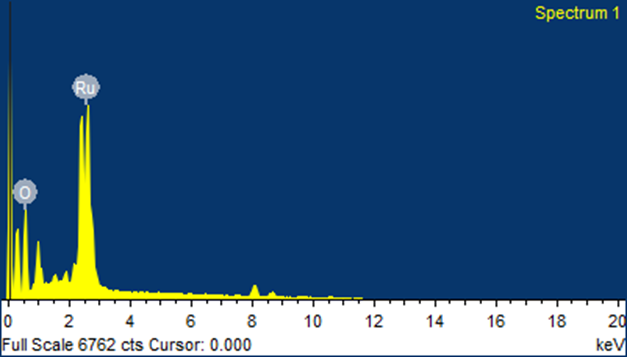 | Element | Weight% |
| --- | --- | --- |
|  | Oxygen | 49.81 |
|  | Ru | 50.19 |
|  | Total | 100 |
| Fig. S2A HRO | | |
| 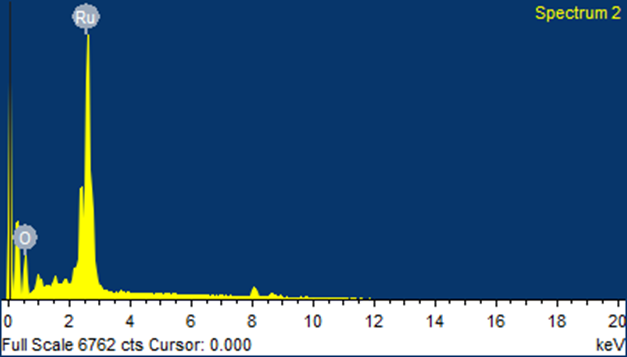 | Oxygen | 28.69 |
|  | Ru | 71.31 |
|  | Total | 100 |
| Fig. S2B Ru-HRO-2 | | |
| Fig. S2 SEM-EDX of materials | | |

|  |
| --- |
| Fig. S3 Proposed mechanism for sugar to sugar alcohol using HRO/Na-β pre-catalyst |

| Table S2 Recyclability studies of Ru-HRO/Na-β*^a^* | | |
| --- | --- | --- |
|  | | |
| Entry | Catalyst/Pre-catalyst | Conv. (%) |
| 1 | HRO/Na-β | 100 |
| 2 | Ru-HRO/Na-β-1 | 100 |
| 3 | Ru-HRO/Na-β-2 | >99 |
| 4 | Ru-HRO/Na-β-3 | >99 |
| 5 | Ru-HRO/Na-β-4 | 98 |
| 6 | Ru-HRO/Na-β-5 | 98 |
| *^a^*Reaction conditions: 1 g of xylose in 40 ml of H_2_O, 50 mg of HRO/Na-β pre-catalyst (5 wt% of Ru) , 80 °C, 20 bar H_2_, 30 min. | | |

| Table S3 Effect of reaction temperature and pressure using HRO/Na-β pre-catalyst | | | | |
| --- | --- | --- | --- | --- |
| S.No | Temp. (°C) | H_2_ pressure  (in bar) | Time (min) | Conv. (%) |
| 1 | 100 | 20 | 10 | 62 |
| 2 | 200 | 20 | 10 | 100 |
| 3 | 100 | 40 | 10 | 100 |
| Reaction conditions: 1 g of glucose in 40 ml of H_2_O, 50 mg of HRO/Na-β pre-catalyst (5 wt%). | | | | |
